# BBC3 in macrophages promoted pulmonary fibrosis development through inducing autophagy during silicosis

**DOI:** 10.1038/cddis.2017.78

**Published:** 2017-03-09

**Authors:** Haijun Liu, Yusi Cheng, Jian Yang, Wei Wang, Shencun Fang, Wei Zhang, Bing Han, Zewei Zhou, Honghong Yao, Jie Chao, Hong Liao

**Affiliations:** 1 Neurobiology Laboratory, New Drug Screening Centre, China Pharmaceutical University, Nanjing, China; 2 Department of Physiology, School of Medicine, Southeast University, Nanjing, China; 3 Nine Department of Respiratory Medicine, Nanjing Chest Hospital, Nanjing, China; 4 Department of Pharmacology, School of Medicine, Southeast University, Nanjing, China; 5 Key Laboratory of Developmental Genes and Human Disease, Southeast University, Nanjing, China; 6 Department of Respiration, Zhongda Hospital, School of Medicine, Southeast University, Nanjing, China

## Abstract

Following inhalation into the lungs, silica particles are engulfed by alveolar macrophages, which triggers endogenous or exogenous apoptosis signaling pathways. As an inducer of apoptosis, the role of BBC3/PUMA (BCL2-binding component 3) in macrophages during silicosis remains unknown. Here, we exposed U937 cell-derived macrophages (UDMs) to SiO_2_ *in vitro* to explore the function of BBC3 in SiO_2_-induced disease. We found that SiO_2_ induced increased BBC3 expression, as well as macrophage activation and apoptosis. Knockdown of *Bbc3* with specific siRNA significantly mitigated the SiO_2_-induced effects. In addition, our results clearly showed increased levels of autophagy in macrophages exposed to SiO_2_. However, inhibition of BBC3 decreased the occurrence of autophagy. Furthermore, we observed that the blockade of autophagy with 3-MA, an autophagy inhibitor, inhibited SiO_2_-induced macrophage activation and apoptosis. In contrast, rapamycin, an autophagy inducer, further enhanced the effects induced by SiO_2_. The conditioned medium from macrophages exposed to SiO_2_ promoted the proliferation and migration of fibroblasts, and the inhibition of BBC3/autophagy reduced the effects of the conditioned medium on fibroblasts. In the mouse model of silicosis, *Bbc3* knockout mice clearly exhibited decreased levels of autophagy and fibrosis progression. These results suggest that downregulation of BBC3 expression may become a novel therapeutic strategy for the treatment of silicosis.

Silicosis is caused by inhaling silica dust (SiO_2_), which is globally prevalent, fatal, and characterized by collagen deposition and myofibroblast hyperplasia.^1^ Many individuals suffering from this condition experience great physical pain and heavy economic burdens.^2^ Thus, there is an urgent need to explore the pathogenesis of silicosis and develop effective therapeutic strategies.

Several studies have shown that alveolar macrophage activation and apoptosis play vital roles in silicosis.^4,3^ Following phagocytosing silica particles, damaged macrophages secrete various factors and deliver different signals to neighboring cells, including fibroblasts and macrophages. The signaling could promote the fibroblast proliferation and eventually lead to pulmonary fibrosis.^5, 6^ Macrophages are considered to have great plasticity in function and phenotype.^7^ Macrophage activation may occur in response to of diverse external stimuli; activated macrophages differentiate into distinct subpopulations, including proinflammatory/cytotoxic M1 and anti-inflammatory/pro-fibrotic M2 macrophages.^8^ These different phenotypes may play distinct roles in different types of immune responses both *in vivo* and *in vitro*.^9^ It has been reported^10^ that the transformation of alveolar macrophages into M1 and M2 phenotypes is associated with the development and progression of silicosis; however, the precise etiology of this mechanism remains to be explored.

A molecule known as TP53-upregulated modulator of apoptosis (BBC3/PUMA) is a member of the BCL2 family that contains a BCL2-like domain. As a potent activator of apoptosis, BBC3 is expressed in multiple cells, such as neuronal, intestinal, and immune cells, and is involved in a variety of pathological processes.^11,12^ Recent studies^13^ have demonstrated that BBC3 signaling mediates ROS production, DNA damage-dependent cell cycle arrest and caspase-independent apoptosis in macrophages through mitochondrial pathways, underscoring the potential role of BBC3 in the progression of some diseases caused by macrophage dysfunction. Autophagy is a process that has been highly conserved evolutionarily and is influential in cellular development. During autophagy, intracellular proteins and organelles are engulfed by autophagosomes, degraded and recycled.^14^ However, a growing number of studies^15, 16^ have revealed that in cells under stress associated with tumor promotion, autophagy was suppressed by tumor promoters and promoted by tumor suppressors, suggesting that autophagy may function as a negative regulator for cell survival.

Some studies^17^ have shown the ability of BBC3 to induce autophagy through BAX activation and mitochondrial outer membrane permeabilization, which further enhanced apoptosis. Furthermore, the cross-talk between autophagy and apoptosis has been extensively studied and shown to affect many physiological and pathological processes.^18, 19^ In view of existing studies, we investigated the impact of BBC3 on macrophages differentiated from human leukemic U937 cells. We found that BBC3 induced macrophage autophagy and further led to cell activation and apoptosis in an *in vitro* model induced by SiO_2_. Furthermore, the conditioned medium obtained from macrophages promoted the activation and migration of lung fibroblasts. These findings provide evidence that increased BBC3 expression and further enhancement of macrophage autophagy are involved in the development of silicosis, which improves the understanding of the link between BBC3 and silicosis.

## Results

### SiO_2_ induced the activation and apoptosis of macrophages differentiated from U937 cells

**Figure 1:**
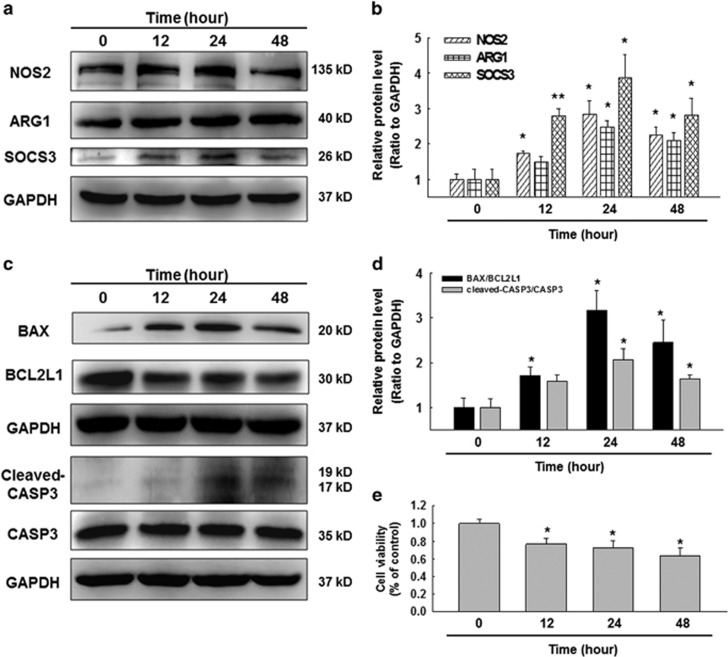
SiO_2_ induced the activation and apoptosis of U937 cell-derived macrophages. (**a**–**d**) Representative Western blot and densitometric analyses showing the effects of SiO_2_ on the expression of the M1 marker NOS2, the M2a marker ARG1, the M2c marker SOCS3 (**a** and **b**) and the apoptosis-associated proteins, BAX, BCL2L1 and cleaved-CASP3 (**c** and **d**) in U937 cells. The results suggested that SiO_2_ induced NOS2, ARG1, SOCS3, BAX, BCL2L1 and cleaved-CASP3 expression in a time-dependent manner. Data are presented as the mean±S.E.M. (*n*=5); *P<0.05; **P<0.01 *versus* the 0-h group (two-way ANOVA). (**e**) MTT assay results showing that the SiO_2_-induced decrease in cell viability occurred in a time-dependent manner in U937 cells. Data are presented as the mean±SEM (*n*=5); *P<0.05 *versus* the 0-h group (Student's *t*-test)

Macrophage polarization plays an important role in the etiology of pulmonary fibrosis.^20^ In this study, to evaluate whether SiO_2_ administration resulted in the activation and apoptosis of U937-derived macrophages (UDMs), western blot was performed to detect M1 and M2 marker proteins, including NOS2 (iNOS), ARG1 (Arginase 1), and SOCS3 (suppressor of cytokine signaling 3). Our results showed that NOS2, ARG1, and SOCS3 were all significantly increased in macrophages in response to SiO_2_ compared with the control group, and all the expression levels peaked at 24 h (Figures 1a and b).

A previous study reported that SiO_2_ induced alveolar macrophage apoptosis by extrinsic pathways in silicosis;^3^ however, whether UDMs would also undergo apoptosis after treatment with SiO_2_ remained unknown. In this study, the Western blot results showed that SiO_2_ caused markedly aggravated apoptosis of these cells (Figures 1c and d). The MTT results further verified that SiO_2_ reduced macrophage viability and promoted cell apoptosis (Figure 1e).

### Activated BBC3 in macrophages exposed to silica mediated activation and apoptosis

**Figure 2:**
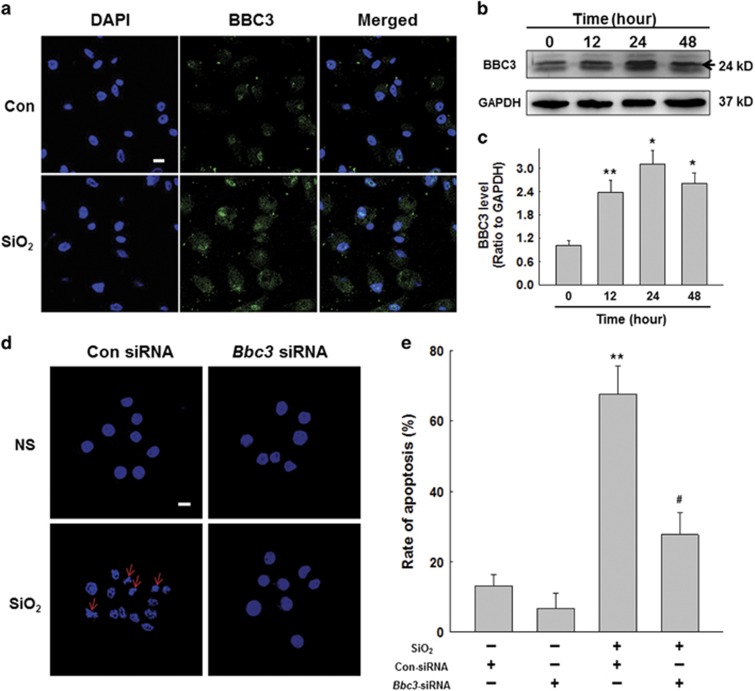
Activated BBC3 in macrophages exposure to silica mediated cell apoptosis. (**a**) Representative immunocytochemical images showing that SiO_2_ increased the expression of BBC3 (green) in U937 cells. Scale bar=10 *μ*m. Images are representative of three independent experiments. (**b** and **c**) Representative Western blot and densitometric analyses showing the effects of SiO_2_ on the expression of BBC3 in U937 cells, suggesting that SiO_2_ induced BBC3 expression in a time-dependent manner. Data are presented as the mean±SEM (*n*=5); *P<0.05; **P<0.01 *versus* the control group (Student's *t*-test). (**d** and **e**) Representative images and data of Hoechst 33342 staining demonstrating that the apoptosis of U937 cells induced by SiO_2_ was attenuated by *Bbc3*-specific siRNA. Scale bar=10 *μ*m. Data are presented as the mean±S.E.M. Images are representative of five independent experiments, and approximately 100 cells were counted in each experiment; **P<0.01 *versus* the con-siRNA group; ^#^*P*<0.05 *versus* the con-siRNA+SiO_2_ group (two-way ANOVA)

Recent studies^21^ have indicated that BBC3 is involved in the pathogenesis of several fibrosis diseases. Therefore, we postulated that BBC3 may participate in the development of silicosis, and we conducted experiments to assess whether BBC3 changes were associated with the activation and apoptosis of macrophages exposed to SiO_2_. Immunofluorescence analysis showed significantly increased expression of BBC3 in macrophages in response to SiO_2_ (Figure 2a). The Western blot results also showed enhanced BBC3 protein expression in macrophages treated with SiO_2_, which reached its peak at 24 h (Figures 2b and c). Figures 2d, e and Supplementary Figure S1A–B showed that *Bbc3* knockdown in macrophages was accompanied by attenuated macrophage activation and apoptosis induced by SiO_2_.

### BBC3-mediated autophagy processes in UDMs exposed to silica

**Figure 3:**
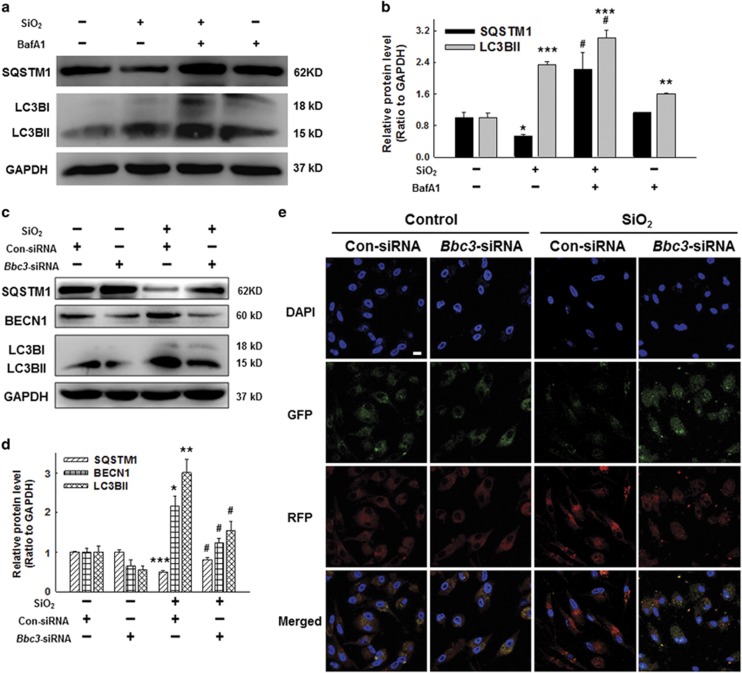
BBC3-mediated autophagy processes in U937-differentiated macrophages exposed to silica. (**a** and **b**) Macrophages were pretreated with or without BafA1 (50 nM) (**b**) for 1 h followed by SiO_2_ treatment for 12 h. Representative Western blot and densitometric analyses showing that bafilomycin A1 (BafA1) further increased accumulation of LC3BII and SQSTM1 induced by SiO_2_. Data are presented as the mean±SEM (*n*=3); *P<0.05; **P<0.01; ***P<0.001 *versus* the control group; ^#^*P*<0.05 *versus* the SiO_2_ group (Student's *t*-test). (**c** and **d**) Representative Western blot and densitometric analyses showing the effects of *Bbc3*-specific siRNA on the expression of BECN1 and LC3B induced by SiO_2_. The results suggest that SiO_2_ induced BECN1 and LC3BII expression, but reduced SQSTM1 expression, which were reversed by the *Bbc3*-specific siRNA. Data are presented as the mean±SEM (*n*=3); *P<0.05; **P<0.01; ***P<0.001 *versus* the con-siRNA group; ^#^*P*<0.05 *versus* the con-siRNA+SiO_2_ group (two-way ANOVA). (**e**) U937-differentiated macrophages were transfected for 12 h with fluorescent mRFP-GFP-tagged LC3 plasmids and then treated with *Bbc3*-specific siRNA or non-specific siRNA using Lipofectamine 2000, followed by treatment with SiO_2_ for 24 h. Images were captured by confocal microscopy. The numbers of red, green, and yellow puncta were analyzed to evaluate autophagic flux, and more than 10 cells were quantified for each condition. SiO_2_ significantly induced autophagy (yellow and red puncta), but *Bbc3*-specific siRNA reversed this effect. Scale bar=10 *μ*m. Images are representative of five independent experiments

Autophagy has been shown^22^ to be dramatically enhanced in rodent models of silicosis. However, the involvement of autophagy in UDMs in response to SiO_2_ *in vitro* remains to be elucidated. In previous studies, THP-1 cells showed enhanced autophagy followed by PMA treatment. Therefore, we examined the effect of PMA alone on autophagy level of U937 cells, and found that there was no significant difference in the expression of autophagy-associated proteins, including LC3B (MAP1LC3B) and SQSTM1/sequestosome 1, between PMA and control group (Supplementary Figure S2A and B). The contradictory conclusions may be result from the stabilization period for U937 cells after PMA treatment that allowed the autophagy activity of U937 cells return to normal level. Next, the western blot results showed that both LC3BII and BECN1/beclin 1 expression levels were significantly increased after silica treatment (Supplementary Figure S2A and B). Consistently, expression of SQSTM1, a receptor protein that links LC3B with ubiquitin moieties on misfolded proteins, was downregulated by silica treatment (Supplementary Figure S2A and B). Our results also showed increased accumulation of LC3BII and SQSTM1 when autophagy flux was blocked with the autophagosome-lysosome fusion inhibitors, bafilomycin A1 (BafA1; Figures 3a and b).

We then assessed whether BBC3 downregulation might be complicit in changes in the autophagic process. The results showed that the knockdown of *Bbc3* with specific siRNA significantly decreased the expression of LC3BII and BECN1, but increased the expression of SQSTM1 compared with the scrambled siRNA control group (Figures 3c and d). The mRFP-GFP-LC3 adenoviral transfection assay results were collected using laser scanning confocal microscopy and showed greater red fluorescence in cells treated with SiO_2_ than in control cells. However, a significantly weakened autophagic flux in macrophages resulting from BBC3 loss was clearly observed (Figure 3e).

### *Bbc3* Knockout reduced autophagy in mouse in response to silica exposure

**Figure 4:**
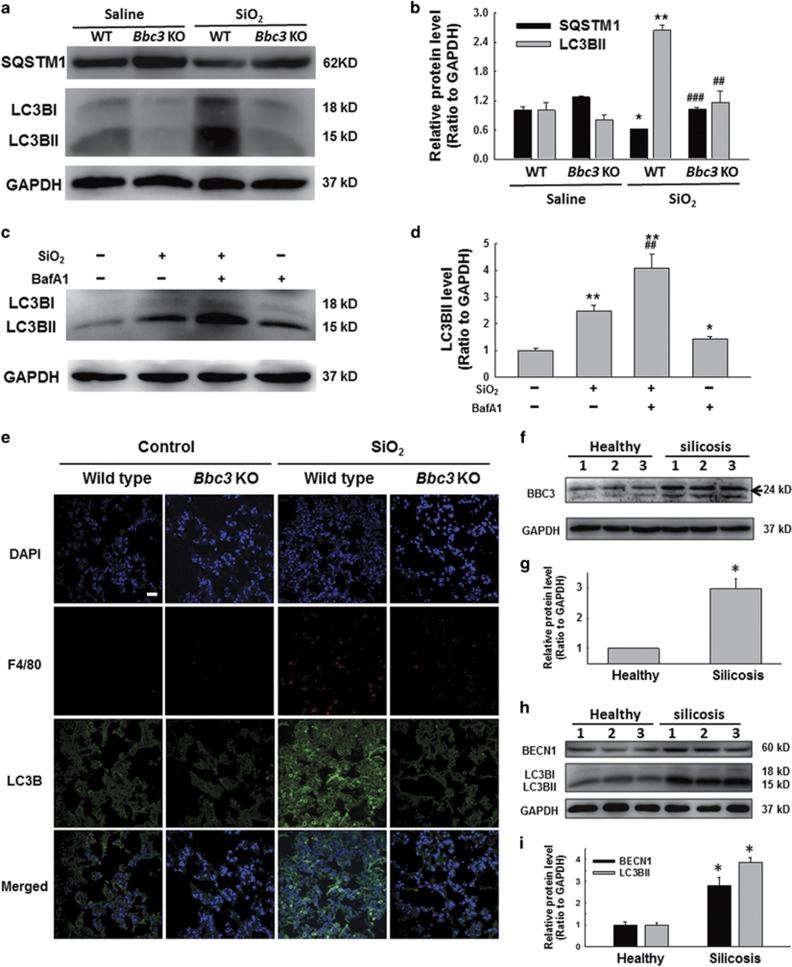
BBC3-mediated autophagy processes in mouse silicosis model. (**a** and **b**) Representative Western blot and densitometric analyses showing that *Bbc3* knockout decreased the LC3BII expression, but increased the SQSTM1 expression in bone marrow-derived macrophages (BMDMs) exposed to SiO_2_. Data are presented as the mean±S.E.M. (*n*=3); *P<0.05; **P<0.01 *versus* the WT group; ^##^*P*<0.01; ^###^*P*<0.001 *versus* the WT+SiO_2_ group (two-way ANOVA). (**c** and **d**) Representative Western blot and densitometric analyses showing that BafA1 further increased accumulation of LC3BII induced by SiO_2_ in BMDMs. Data are presented as the mean±SEM (*n*=3); *P<0.05; **P<0.01 *versus* the control group; ^##^*P*<0.01 *versus* the SiO_2_ group (two-way ANOVA). (**e**) Immunohistochemical staining of WT and *Bbc3* KO mouse lung tissue showing autophagy intensity. The results indicated that the loss of BBC3 reduced the number of macrophages and the expression of SQSTM1 in lung tissue sections. Scale bar=20 *μ*m. Images are representative of several individuals from each group (*n*=4). (**f**) Representative western blot showing the expression of BBC3 in macrophages from healthy donors and silicosis patients. (**g**) Densitometric analyses of macrophage samples from five healthy donors and five silicosis patients suggested that BBC3 expression was elevated in macrophages from silicosis patients. Data are presented as the mean±S.E.M.; *P<0.05 *versus* the corresponding healthy control group (Student's *t*-test). (**h**) Representative western blot showing the expression of BECN1 and LC3B in macrophages from healthy donors and silicosis patients. (**i**) Densitometric analyses of macrophage samples from five healthy donors and five silicosis patients suggested that BECN1 and LC3BII expression was elevated in macrophages from silicosis patients. Data are presented as the mean±SEM; *P<0.05 *versus* the corresponding healthy control group (Student's *t*-test)

Then, we further performed our experiments in bone marrow-derived macrophages (BMDMs). MTT results showed that SiO_2_ reduced the BMDM viability in a time-dependent manner (Supplementary Figure S3A). Then, western blot results indicated that SiO_2_ treatment significantly increased the LC3BII expression, but reduced the SQSTM1 expression in BMDMs (Supplementary Figure S3B and C). Moreover, we also found that *Bbc3* knockout significantly decreased the LC3BII expression induced by SiO_2_ in BMDMs; however, the decrease of SQSTM1 expression caused by SiO_2_ exposure in BMDMs was alleviated in BMDMs from *Bbc3* knockout mice (Figures 4a and b). To examine whether the increase of LC3BII was due to enhanced autophagy initiation or inhibition of lysosomal degradation, BMDMs were pretreated with BafA1 for 1 h followed by SiO_2_ treatment. Our results showed that BafA1 further increased the LC3BII expression induced by SiO_2_ in BMDMs (Figures 4c and d), suggesting that SiO_2_ increased LC3BII levels primarily through enhancing autophagy initiation.

We then established a mouse silicosis model to conduct further experiments. The immunohistochemical staining results showed a significant increase of macrophages in quantity and autophagy level as indicated by enhanced LC3B expression and reduced SQSTM1 expression in WT mice treated with SiO_2_ suspension for 1 month compared with that of mice in the control group; however, *Bbc3* knockout decreased the autophagy levels in the silicosis model (Figure 4e and Supplementary Figure S3D).

To further determine whether the autophagic protein regulation of BBC3 was associated with human silicosis, we examined the expression of BBC3 and autophagy proteins in macrophages from the BALF of normal subjects and silicosis patients. As shown in Figures 4f and g, compared with normal individuals, silicosis patients exhibited significantly increased BBC3 levels in BALF macrophages. Furthermore, the expression of LC3BII and BECN1, two autophagic markers, were also increased in macrophages from the BALF of silicosis patients (Figures 4h and i). Collectively, these results suggest that BBC3 contributes to the regulation of autophagy protein expression during silicosis.

### Autophagy is responsible for macrophage activation and apoptosis in response to silica

**Figure 5:**
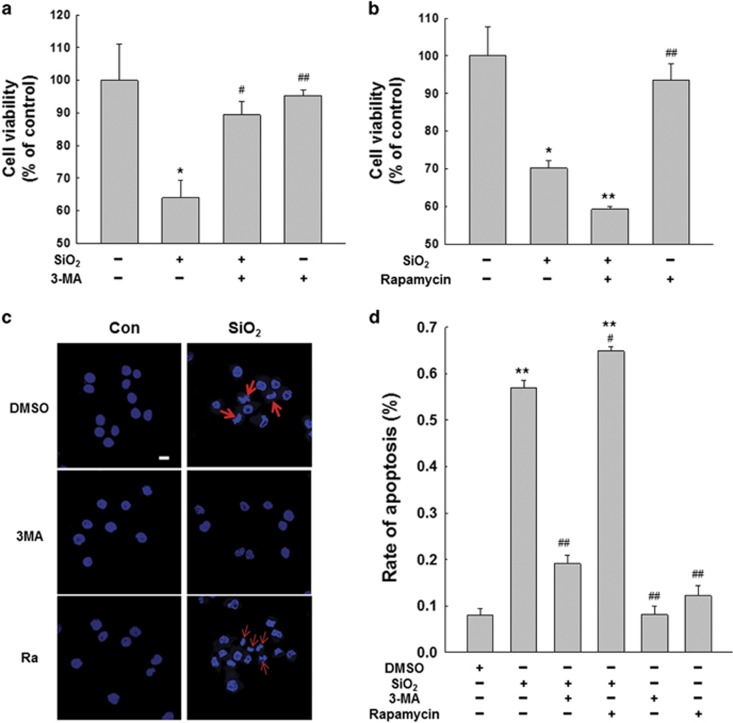
Autophagy is responsible for macrophage apoptosis in response to silica. MTT assay results showing that 3-MA mitigated the decrease in cell viability induced by SiO_2_ (**a**); however, rapamycin aggravated the decrease (**b**). Data are presented as the mean±SEM (*n*=5); *P<0.05; **P<0.01 *versus* the control group; ^#^*P*<0.05; ^##^*P*<0.01 *versus* the SiO_2_ group (two-way ANOVA). (**c** and **d**) Representative images of Hoechst 33342 staining demonstrating that the apoptosis of U937 cells induced by SiO_2_ was attenuated by 3-MA but was further enhanced by rapamycin. Scale bar=10 *μ*m. Data are presented as the mean±S.E.M. (*n*=5); **P<0.01 *versus* the control group; ^#^*P*<0.05; ^##^*P*<0.01 *versus* the SiO_2_ group (two-way ANOVA)

To determine the role of macrophage autophagy in silicosis, we proceeded to measure the effects of autophagy on macrophage activation and apoptosis followed by SiO_2_ treatment utilizing 3-methyladenine (3-MA) and rapamycin, which inhibit and induce autophagy, respectively. As shown in Supplementary Figure S4A–D, 1 mM of 3-MA significantly inhibited the expression of autophagic proteins, including LC3BII and BECN1, but there is no significant promotion in the expression of autophagic proteins with 1 *μ*M rapamycin compared with cells only treated with SiO_2_. Our results showed that 3-MA significantly dampened the activation and apoptosis of macrophages exposed to SiO_2_ (Supplementary Figure S4E–F). However, there is no significant influence on the activation and apoptosis of macrophages exposed to SiO_2_ following rapamycin treatment (Supplementary Figure S4G–H). The MTT assay results showed that 3-MA reversed the cell viability decrease caused by SiO_2_ (Figure 5a). However, rapamycin further reduced the viability of macrophages treated with SiO_2_ (Figure 5b). Hoechst 33342 staining showed results similar to those of the MTT assay; 3-MA decreased SiO_2_-induced macrophage apoptosis, but rapamycin enhanced the effects of SiO_2_ (Figures 5c and d). These results demonstrated that BBC3 promoted autophagy in macrophages exposed to SiO_2_. We then performed an experiment to obtain more information on the involvement of autophagy in the BBC3-mediated activation and apoptosis of macrophages exposed to SiO_2_. The results indicated that the enhancement of autophagy by rapamycin counteracted the decreased activation and apoptosis of macrophages exposed to SiO_2_ as a result of *Bbc3* inhibition with siRNA (Supplementary Figure S4I and J). These results suggest the possibility of a functional coupling of enhanced autophagy during silicosis and increased macrophage activation and apoptosis, as well as decreased cell viability.

### *Bbc3* RNAi in macrophages attenuated pro-fibrogenic effects of conditioned medium on fibroblasts

**Figure 6:**
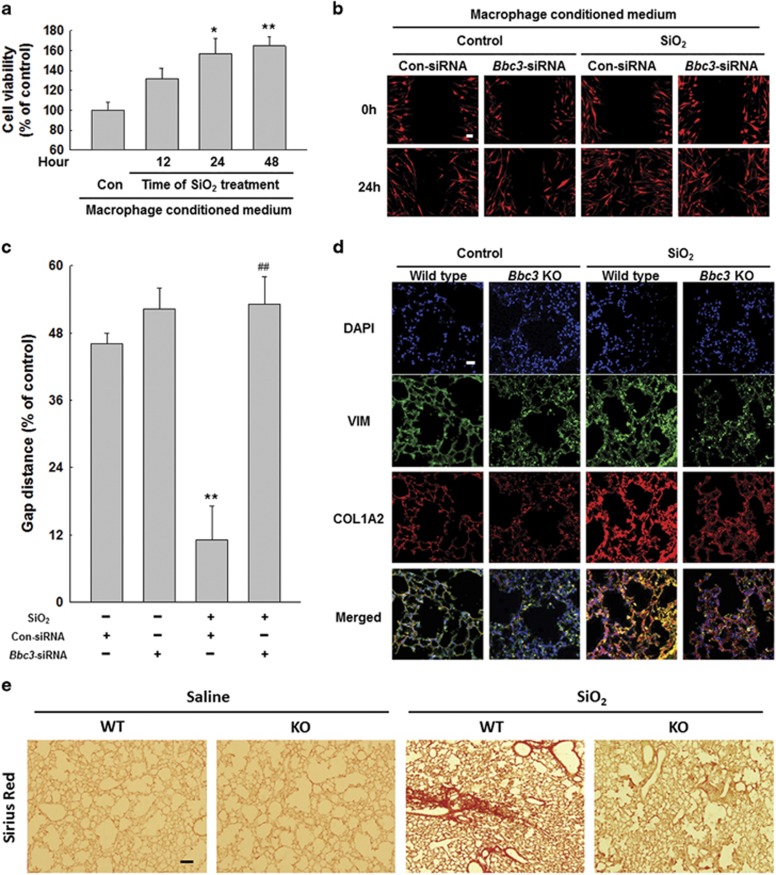
*Bbc3* RNAi in macrophages attenuated pro-fibrogenic effects of conditioned medium on fibroblasts. (**a**) MTT assay results showing that the macrophage-conditioned medium from different time points following treatment with SiO_2_ induced an increase in cell viability in HPF-a cells. Data are presented as the mean±SEM; Experiments were repeated independently five times; *P<0.05; **P<0.01 *versus* the control group (Student's *t*-test). (**b** and **c**) Representative images and data showing the effects of macrophage-conditioned medium on the migration of RFP-labeled HPF-a cells. The results indicated that conditioned medium from macrophages treated with SiO_2_ induced an increase in HPF-a cell migration. However, *Bbc3*-specific siRNA reduced the effects of macrophage-conditioned medium on HPF-a cells. Scale bar=80 *μ*m. Quantification of the change in the scratch gap distance from six separate experiments. Data are presented as the mean±S.E.M.; **P<0.01 *versus* the con-siRNA group; ^##^*P*<0.01 *versus* the con-siRNA+SiO_2_ group (two-way ANOVA). (**d**) Dual immunohistochemical staining of vimentin (VIM) and COL1A2 in the lung tissue of WT and *Bbc3* KO mice. Lung tissue with silicosis exhibited significantly more-intense COL1A2 expression than did control lung tissue, but the loss of BBC3 alleviated the fibrosis, as indicated by reduced COL1A2 staining intensity. Scale bar=20 *μ*m. Images are representative of several individuals from each group (*n*=4). (**e**) Sirius Red staining results showing that *Bbc3* knockout significantly alleviated pulmonary fibrosis compared with control group treated with SiO_2_. Scale bar=50 *μ*m. Images are representative of several individuals from each group (*n*=4)

To begin addressing whether macrophage dysfunction during silicosis stimulates the fibrogenic actions of lung myofibroblasts, we examined the effects of conditioned medium from macrophages exposed to SiO_2_ *in vitro* on fibroblast behavior. As shown in Supplementary Figure S5A and B, after pulmonary fibroblasts were cultured with the conditioned medium for 24 h, we observed decreased expression of BAX and increased protein expression of ACTA2/*α* smooth muscle actin, collagen and BCL2L1. The MTT assay results indicated that conditioned medium from macrophages treated with SiO_2_ for different times improved fibroblast viability (Figure 6a). Then, further analysis showed that, following fibroblasts cultured with the conditioned medium, the BBC3 inhibition with *Bbc3*-specific siRNA significantly decreased the expression of fibrosis-associated proteins, including COL1A2/collagen I, COL3A1/collagen III, ACTA2 and BCL2L1, but increased BAX protein expression (Supplementary Figure S5C and D). The cell migration assay showed that BBC3 knockdown in macrophages notably reduced the effect of conditioned medium from macrophages exposed to SiO_2_ on fibroblast migration compared with that of the control (Figures 6b and c). We then examined the degree of fibrosis in *Bbc3* KO mice treated with SiO_2_, and we found that COL1A2 expression was significantly lower in the *Bbc3*^−/−^ mice than in WT mice (Figure 6d). Furthermore, Sirius Red staining results also indicated that *Bbc3 knockout* significantly alleviated pulmonary fibrosis compared with WT group treated with SiO_2_ (Figure 6e). Collectively, these results suggest that increased BBC3 expression in macrophages is causally linked to fibroblast activation and migration during silicosis.

### SiO_2_-induced autophagy in macrophages is involved in HPF-a cell activation and migration

**Figure 7:**
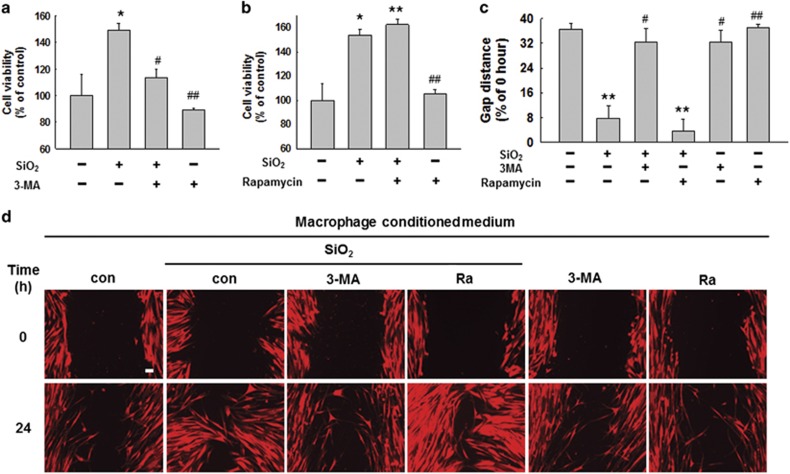
SiO_2_-induced autophagy in macrophages is involved in HPF-a cell activation and migration. MTT assay results showing that the pretreatment of U937 cells with 3-MA attenuated the increase in fibroblast viability induced by macrophage-conditioned medium (**a**), but the pretreatment of U937 cells with rapamycin further promoted the effects of macrophage-conditioned medium induced by SiO_2_ (**b**). Data are presented as the mean±SEM (*n*=5); *P<0.05; **P<0.01 *versus* the control group; ^#^*P*<0.05; ^##^*P*<0.01 *versus* the SiO_2_ group (two-way ANOVA). (**c** and **d**) Representative images and data showing the effects of the macrophage-conditioned medium on the migration of RFP-labeled HPF-a cells. The pretreatment of U937 cells with 3-MA attenuated the pro-migration effects induced by SiO_2_ of macrophage-conditioned medium on fibroblasts; however, rapamycin aggravated the effects. Scale bar=80 *μ*m. Quantification of the change in scratch gap distance from six separate experiments. Data are presented as the mean±SEM; **P<0.01 *versus* the control group; ^#^*P*<0.05; ^##^*P*<0.01 *versus* the SiO_2_ group (two-way ANOVA)

We then explored the role of macrophage autophagy in fibroblast activation and migration. As shown in Supplementary Figure S6A and B, conditioned medium from macrophages treated with 3-MA prior to SiO_2_ exposure resulted in decreased fibroblast activation and proliferation, as indicated by decreased expression of ACTA2, collagen and BCL2L1, as well as increased BAX expression. However, the conditioned medium from macrophages treated with rapamycin resulted in increased fibroblast activation and proliferation (Supplementary Figure S6C and D). Further study showed that the inhibition of macrophage autophagy with 3-MA significantly decreased the effects of conditioned medium from macrophages exposed to SiO_2_ on fibroblast viability and migration; however, rapamycin increased the promotive effects of conditioned medium on fibroblast viability and migration (Figures 7a–d).

## Discussion

Alveolar macrophages respond to particles inhaled through the pulmonary bronchial airway via intricate interactions with other cells, such as fibroblasts and epithelial cells. These macrophages function as effector cells by secreting and releasing factors that attract and regulate other cells, resulting in the ever-increasing presence of mesenchymal components.^23^

BBC3, a downstream molecule of TP53, functions as a pro-apoptosis protein and has a prominent role in a variety of pathological conditions. BBC3 has been reported to regulate BAX activation induced by oxidative stress and neuronal apoptosis.^24^ Furthermore, BBC3 can mediate apoptosis by directly binding BCL2 and BCL2L1 via its BH3 domain, replacing BAX.^25^ In our earlier work,^26^ we found that TP53 and BBC3 protein expression rapidly and persistently increased in pulmonary fibroblasts exposed to SiO_2_; in addition, the knockdown of *TP53* and *Bbc3* with specific siRNA impaired fibroblast activation and migration caused by SiO_2_ exposure. These results underlined the pivotal role of BBC3 in silicosis development. In this study, we exposed UDMs to SiO_2_ and evaluated the influence of BBC3 on the pro-fibrogenic effects induced by SiO_2_. As expected, we found that BBC3 was significantly increased in macrophages exposed to SiO_2_ in a time-dependent manner.

In response to different stimuli, macrophages can differentiate into different phenotypes, such as M1 and M2.^27^ M1 macrophages are associated with inflammation. In contrast, M2 macrophages are associated with enhanced proline and polyamine biosynthesis, which indicates an important role in cell proliferation, tissue repair and collagen production.^27^ The present study showed that SiO_2_ induced dramatically increased protein expression of NOS2 (M1 marker), SOCS3, and ARG1 (M2 marker) in macrophages, suggesting that both activation signals are involved in the SiO_2_-induced pathology. In contrast with previous studies,^28, 29^ the present study found that M1 and M2 marker proteins were both greatly increased in the UDMs, *versus* transforming into either M1 or M2. We speculated that the U937 cells at least partially differentiated into M0 macrophages^30, 31^ following the PMA treatment and that these M0 macrophages further transformed into M1 and M2 macrophages in response to the SiO_2_.

The phagocytosis of cytotoxic silica dust by alveolar macrophages causes mitochondrial damage that leads to a dramatic loss in mitochondrial transmembrane potential and transient phagolysosomal leakage of its contents into cytoplasm, which initiates various apoptotic signaling pathways. A large body of evidence supports the idea that excessive cell death mediated by apoptosis is involved in the etiology of silicosis.^16, 32^ In this context, both *in vitro* and *in vivo* studies have shown decreased viability of alveolar macrophages and cell lines.^33^ However, treatment of these cells with pan-caspase inhibitor z-VAD-fmk dramatically reduced both cell death and collagen deposition, which indicated the possibility that apoptosis plays an important role in contributing to the progression of silicosis.^34^ In this study, we found that exposing macrophages to SiO_2_ resulted in increased expression of apoptosis-associated proteins and reduced anti-apoptotic protein expression. Furthermore, we found that apoptosis was also involved in the pathogenesis of silicosis due to the dysfunction of UDMs. Subsequently, our results demonstrated that treating the macrophages with *Bbc3*-specific siRNA remarkably inhibited cell activation and apoptosis, suggesting that silicosis resulting from macrophage activation and apoptosis may depend on the upregulation of BBC3. Furthermore, we found that there was a basal decrease in the expression of marker proteins in macrophages in response to *Bbc3*-siRNA treatment. It was supposed that BBC3 could regulate macrophage activation even at the basal level in UDMs. However, SiO_2_-induced BBC3 upregulation in macrophages caused dysregulation of BBC3 on macrophage activation that led to increased expression of marker proteins. These results indicate that the regulation of BBC3 on macrophage activation is effective in more extensive conditions.

Autophagy is an important pro-survival mechanism for maintaining metabolic homeostasis under short-term, mild stress; however, apoptosis signaling cascades are initiated following extended, excessive activation of autophagy.^19, 23^ An increasing number of studies have concentrated on the influence of autophagy on fibrosis.^35, 36^ However, the effects of autophagy have been found to be multifaceted in respiratory diseases, including idiopathic pulmonary fibrosis,^37^ cystic fibrosis,^38^ and acute lung injury.^39^ Other studies have observed that autophagic activity was variable in a silicosis rat model.^22^ Meanwhile, increased levels of LC3 and BECN1 have been observed in groups of stage I, II and III patients exposed to SiO_2_ compared with an observer group.^40^ Moreover, exposure to silica nanoparticles in endothelial cells induces inflammatory response, activates autophagy, and eventually leads to endothelial dysfunction in silicosis (ref. 41). A recent study^42^ shows that silica (mean particle diameter 1.5–2 *μ*m) exposure causes increased expression of LC3-II and SQSTM1 *in vitro* and isolated Alveolar Macrophages (AM) from silica-exposed mice, indicating increased autophagy. Moreover, BafA1 treatment of silica-exposed cells leads to further increases in LC3-II and SQSTM1 expressions compared with BafA1-treated control. These findings indicate that autophagy is increased but not necessarily impaired following silica exposure. Meanwhile, this study also shows that autophagy deficiency in Atg5^fl/fl^LysM-Cre^+^ mice enhances silica-induced cell death and inflammation. The inconsistent results from ours may be caused by differences in the particle diameter (mean particle diameter 2–5 *μ*m in our study) and administration mode of SiO_2_. In addition, autophagy is critical in maintaining cell homeostasis. Both deficient and excessive autophagy will lead to the destruction of cell homeostasis.^18, 43^ In our present study, SiO_2_ exposure induced excessive autophagy, resulted in cell dysfunction and ultimately caused pulmonary fibrosis. But despite all this, many studies by others and us suggested that autophagy played a vital role in pulmonary fibrosis.^42, 44^

Some evidence^17^ has shown that BBC3, as a BH3-only protein, triggered autophagic responses depending on the concurrent presence of BAX or BAK1 with apoptosis. In the present study, our results showed that the knockdown of *Bbc3* expression with specific siRNA resulted in surprisingly inhibited autophagy. Furthermore, we also found that after being exposed to an SiO_2_ suspension by intratracheal instillation, *Bbc3* KO mice exhibited markedly attenuated autophagy progression compared with that of WT mice. In addition, compared with healthy people, silicosis patients showed significantly increased expression levels of BBC3 and autophagic proteins in BALF macrophages. BBC3 has been speculated to promote the development of silicosis through inducing autophagy. Some studies have revealed that BCL2L1 and BCL2 have been shown to inhibit autophagy by binding to BECN1, an autophagy-inducing protein which contains a BH3 domain.^45^ According to present results, we speculate that BBC3, as a pro-apoptotic BH3-only protein, functions to induce autophagy through competitively disrupting the interaction between BECN1 and BCL2L1.^17^ A separate research^46^ shows that BBC3 is a substrate of chaperone-mediated autophagy (CMA) in human tumor cell lines, but inhibition of CMA results in stabilization of BBC3. These investigations demonstrate that the behavior of BBC3 is complicated and performs various functions depending on cell type and cellular stress stimuli. In this study, the inhibition of autophagy in macrophages with 3-MA significantly abated the activation and apoptosis of macrophages induced by SiO_2_; however, rapamycin enhanced the effects of SiO_2_ on macrophages. These results suggested that BBC3 regulated the activation and apoptosis of UDMs by activating autophagy.

Silicosis is a progressive fibrosis disorder involving interactions among multiple cell types. In the present study, conditioned medium from macrophages exposed to SiO_2_ significantly enhanced the viability and migration of human pulmonary fibroblasts compared with conditioned medium from macrophages not treated with SiO_2_. However, treating macrophages with *Bbc3*-specific siRNA notably decreased the pro-fibrotic effects of the conditioned medium. Similarly, 3-MA also inhibited the fibrosis activity of the conditioned medium; however, rapamycin further enhanced the fibrotic effects. Moreover, in our recent report,^47^ we found that several important pro-fibrotic cytokines, including TGF-*β*, TNF-*α,* and MCP-1, were induced in supernatant of macrophages in response to SiO_2_, however, pretreatment with 3-MA or Z-VAD-FMK, an apoptosis inhibitor, attenuated these changes. These results suggest that the macrophages secreted some mediators into the conditioned medium, thus exerting pro-fibrotic effects. And BBC3 mediated the fibroblast activation and migration through inducing macrophage autophagy and apoptosis, suggesting an important role of BBC3 in macrophages in SiO_2_-induced pulmonary fibrosis.

**Figure 8:**
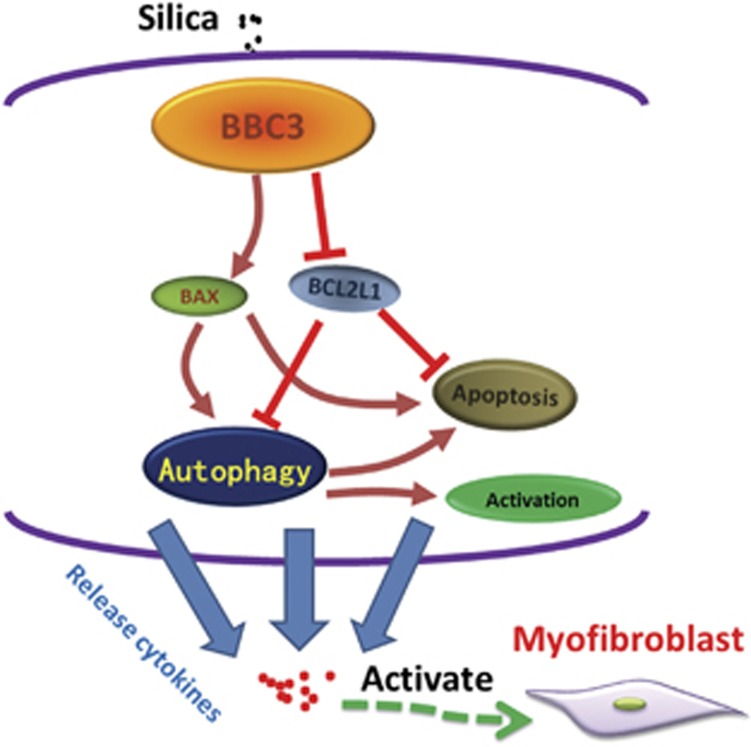
Schematic model of silica-induced pulmonary fibrosis. Silica causes increased expression of BBC3 in U937 cell-derived macrophages, which promotes autophagy by enhancing BAX expression and attenuating BCL2L1 expression. Autophagy is further aggravated by macrophage apoptosis and activation, leading to the release of cytokines from macrophages and the activation of fibroblasts into myofibroblasts. Myofibroblasts participate in the fibrotic process by proliferating, migrating and producing collagen

In summary, the present study highlights that BBC3 significantly increased the activation and apoptosis of macrophages by enhancing autophagy, triggering the release of pro-fibrotic cytokines and improving the viability and migration of human pulmonary fibroblasts (Figure 8). The results indicate that BBC3 is involved in the pathological progress of silicosis. The inhibition of BBC3 and autophagy decreased the activation and apoptosis induced by SiO_2_ in UDMs, which further attenuated the pro-fibrotic activity of conditioned medium from macrophages exposed to SiO_2_ on human pulmonary fibroblasts. This discovery of upregulated expression of BBC3 in the development of pulmonary injury following SiO_2_ exposure will facilitate the research and development of effective therapies for silicosis through targeting against BBC3 and serve as an important tool for the accurate diagnosis of this condition by detection of BBC3 expression.

## Materials and Methods

### Animals

*Bbc3* knockout (KO) mice were obtained from Dr Gerard Zambetti at St. Jude Children's Research Hospital, Inc., and from Dr Tao Cheng from the State Key Laboratory of Experimental Hematology, Institute of Hematology and Blood Disease Hospital, Chinese Academy of Medical Sciences and Peking Union Medical College. The original *Bbc3* KO mice were on a mixed C57BL6/129SV genetic background^48^ and were then backcrossed to C57BL/6 for than more than ten generations (F10). Six- to eight-week old *Bbc3* KO and wild type (WT) littermates were generated from F10 heterozygous breeding. All the animals were male which were housed (4 per cage) in a temperature-controlled room (25 °C, 50% relative humidity) on a 12-h light/dark cycle.

### Reagents

SiO_2_ was purchased from Sigma (S5631) (St. Louis, MO, USA); 80% of particles were less than 5 *μ*m in diameter. The particles were selected by sedimentation according to Stokes' law, acid-hydrolyzed, and baked overnight (200 °C, 16 h). The silica samples for the cell experiments were sterilized by being autoclaved and were then suspended in sterile, normal saline at a concentration of 5 mg/ml. The dosage of SiO_2_ used in this study was based on previous studies.^49, 50^ Due to the inherent insolubility of SiO_2_, it would settle on the bottom of the culture plate; thus, we treated macrophages with SiO_2_ at 50 *μ*g/cm^2^ following seeding at 6 × 10^5^ cells/well in a 24-well plate and allowing cell adherence for 24 h. Fetal bovine serum (FBS, 10099141), normal goat serum (NGS, 31873), Hoechst 33342 (V23201), RPMI 1640 Medium (21875091) and Dulbecco's modified Eagle's medium (DMEM; 11995065) were purchased from Life Technologies (New York, NY, USA). GlutaMax Supplement (35050–061) was obtained from Gibco (New York, NY, USA), and a penicillin/streptomycin mixture (15140–122) was obtained from Fisher Scientific (Waltham, MA, USA). Antibodies against NOS2 (ab3523) was purchased from Abcam (Cambridge, MA, USA). Antibodies against BBC3 (SC374223), BECN1 (SC48341),VIM (SC7558), F4/80 (SC26642), LC3 (SC398822) and GAPDH (SC32233) were obtained from Santa Cruz Biotechnology, Inc (Santa Cruz, CA, USA). The antibody against COL1A2 (BS1530) and COL3A1 (BS1531) were purchased from BioWorld (St. Louis Park, MN, USA). The antibody against SOCS3 (2923S), ARG1(9819S), cleaved-CASP3 (9661S), CASP3 (9662S), BAX (2772S), BCL2L1 (2764S) and LC3 (2775S) were obtained from Cell Signaling, Inc (Beverly, MA, USA). The antibody against ACTA2 (SAB5500002) and Tween-20 were purchased from Sigma-Aldrich (St. Louis, MO, USA). The antibody against SQSTM1 (18420) was obtainted from Proteintech Group (Chicago, IL, USA). The short interfering RNA (siRNA) transfection reagent (SC29528) and *Bbc3* siRNA (SC37153) were purchased from Santa Cruz Biotechnology.

### Establishment of silicosis mouse model

Animals were anesthetized with pentobarbital sodium by intraperitoneal injection, and their tracheae were exposed surgically. Prepared SiO_2_ suspension (0.2 g/kg in 50 mg/ml saline) was instilled intratracheally. The control animals were given the same volume of sterile saline, as previously described.^51^ To collect tissue samples following the administration of SiO_2_ or saline for 1 month, the animals were deeply anesthetized by an overdose of isoflurane, followed by a pneumothorax and perfusion. The pulmonary tissues were dehydrated with 30% sucrose solution and fixed with 4% formalin before being stained. All animal procedures were performed in strict accordance with the ARRIVE guidelines, and the animal protocols were approved by the Institutional Animal Care and Use Committee of the Medical School of Southeast University.

### Cell culture

The human monocyte U937 cell line (ATCC) was grown in RPMI 1640 medium containing 10% FBS with penicillin (50 U/ml) and streptomycin (100 *μ*g/ml) at 37 °C in an incubator with 5% humidified CO_2_ and 95% air. The U937 cells were differentiated into macrophage-like cells by being treated with 50 nM phorbol myristate acetate (PMA, P1585, Sigma-Aldrich) for 24 h.

The human pulmonary fibroblasts (ScienCell, Santa Cruz, CA, USA) were grown in DMEM supplemented with 10% FBS, 100 U/ml penicillin, 100 *μ*g/ml streptomycin and 2 mM l-GlutaMax in a humidified 5% CO_2_ atmosphere at 37 °C.

Bone marrow-derived macrophages (BMDM) were isolated from mouse femurs. Bone marrow was flushed from these bones into 50 ml Falcon tubes with DMEM. Cells were centrifuged and RBCs were lysed by resuspension in 5 ml of Red Blood Cell Lysis Buffer. Finally, cells were cultured in DMEM supplemented with 10% FBS and 20% L929 cell-conditioned medium as previously described.^52,53^ After 3 days of culture, macrophages were detached, counted and plated for subsequent experiments.

### Western blot

After being washed three times with cold phosphate-buffered saline (PBS), treated cells were harvested using a mammalian cell lysis kit (MCL1-1KT, Sigma-Aldrich). Equal amounts of the proteins were subjected to SDS-PAGE (12%) under reducing conditions; the separated proteins were transferred to PVDF membranes, and then blocked with 5% non-fat dry milk in Tris-buffered saline with Tween-20 (TBST) at room temperature for 1 h. The membranes were probed with the indicated antibodies overnight at 4 °C. After three washes, the membranes were incubated with the alkaline phosphatase-conjugated goat anti-mouse/rabbit IgG secondary antibody (1:5000 dilution). A chemiluminescence detection system was used to detect the signals. The intensity of the protein bands was quantified by densitometry using ImageJ software (NIH). Each Western blot was repeated at least three times.

### Scratch assay

To assess the motility of the fibroblasts, the scratch assay was performed as previously described.^54^

### MTT assays

Cell viability was measured using MTT [3-(4,5-dimethylthiazol-2-yl)-2,5-diphenyltetrazolium bromide] assays as previously described.^55^

### siRNA knockdown

RNA interference targeting *Bbc3* was performed to knock down the protein levels of BBC3 in macrophages. In brief, following the differentiation of U937 cells into macrophages with 50 nM PMA, chemically synthesized *Bbc3*-specific siRNA was transfected into the macrophages using Lipofectamine 2000, according to the manufacturer's instructions (Santa Cruz Biotechnology); a non-specific siRNA (SC37007, Santa Cruz Biotechnology) was used as a negative control. The prepared mixture containing siRNA combined with Lipofectamine 2000 was added dropwise to the culture plate. Then, the serum-free DMEM was replaced with complete medium for an additional 24-h incubation prior to subsequent experiments.

### Lentiviral transfection

LV-red fluorescent protein (RFP) lentiviral (Hanbio Inc., Shanghai, CN, USA) transfection was performed using human pulmonary fibroblasts cells (HPF-a), as previously described.^56^ In brief, P3-4 primary HPF-a were seeded at 1 × 10^4^ cells/well in 24-well plates and cultured in DMEM containing 10% FBS for 48 h. The cell medium was replaced with fresh medium mixed with 8 *μ*g/ml polybrene. Subsequently, 100 *μ*l of lentivirus solution (107 IU/ml) was added to each well, and the cells were incubated for 24 h. Then, the treatment medium was replaced with fresh DMEM containing 10% FBS, and the cells were incubated until they reached >50% confluence. The transduced cells were then selected by blasticidin to eliminate uninfected cells as follows: the medium was replaced with fresh medium containing 10 *μ*g/ml puromycin and 10% FBS, and the cells were cultured at 37 °C and 5% CO_2_ for 24 h. Then, the cells were washed twice with fresh medium. Purified, transduced HPF-a cultures were expanded and/or stored in liquid nitrogen, as previously described.^54^

### Detection of autophagic flux

U937 cells were seeded in 24-well plates and differentiated into macrophages via induction by PMA. To detect autophagic flux, the macrophages were transfected with mRFP-green fluorescent protein (GFP)-LC3 adenoviral vectors according to the manufacturer's instructions (Hanbio Inc., Shanghai, CN, USA). Successfully transfected macrophages could express LC3 protein tagged by RFP and GFP. GFP is acid sensitive and shows quenching green fluorescence in the acidic environment of a lysosome. However, in contrast with GFP, RFP is relatively stable within lysosomes. Therefore, the number of GFP and RFP puncta was examined and quantified by confocal microscopy. The 'red' and 'yellow' (i.e., a combination of red and green) spots indicated autophagosomes and autolysosomes, respectively.^57^

### Immunofluorescence staining

Macrophages differentiated from U937 cells were seeded on coverslips, placed in 24-well plates and treated with SiO_2_ for 24 h. Then, the coverslips were rinsed twice with PBS, and the cells were fixed with 4% paraformaldehyde overnight. The fixed cells were incubated with 0.3% Triton X-100 in PBS for 30 min and were then rinsed twice with PBS. The cells were blocked at room temperature for 2 h using 10% NGS in 0.3% Triton X-100 and were then incubated with primary antibodies at 4 °C overnight. After being washed three times with PBS, the cells were incubated with secondary fluorescent antibodies (1:250), and the cell nuclei were then stained with DAPI (4',6-diamidino-2-phenylindole). Images were captured using fluorescent microscopy (Olympus IX70, Olympus America, Inc., Center Valley, PA, USA).

### Hoechst 33342 staining

After being challenged, the macrophages differentiated from U937 cells were washed with DMEM and stained with the DNA-specific dye Hoechst 33342 (5 *μ*g/ml in DMEM) for 10 min in the dark at room temperature. After being washed twice with PBS, the coverslips were mounted on microslides with 50% glycerol and were immediately viewed using a microscope with filters for blue fluorescence. Stained macrophages that exhibited abnormal nuclear condensation and fragmentation were considered apoptotic cells and were counted.

### Sirius red staining assay

For histological analysis of pulmonary fibrosis, the lung was extracted, fixed in 4% formalin and dehydrated in 30% sucrose solution. The sections of the lung were detected using a Sirius Red staining kit (ab150681, Abcam) according to the manufacturer's instructions.

### Human bronchoalveolar lavage fluid

Human BALF was obtained from Nanjing Chest Hospital. Primary alveolar macrophages derived from harvested human BALF were used in accordance with the approved guidelines of the Research and Development Committee of Nanjing Chest Hospital. After being centrifuged at 4 °C for 10 min at 1800 r.p.m., the cells were resuspended in serum-free medium. Then, the cells were seeded in a 24-well plate at 5 × 10^5^ cells/well. After incubating the cells for 2 h at 5% CO_2_ and 37 °C, the serum-free medium was removed from the plate, and the cells were washed with sterile PBS twice to remove non-adherent cells. The remaining adherent cells were continued to be cultured in complete medium for subsequent experiments.

### Statistics

The data are presented as the mean±S.E.M. Significance was established using a *t*-test for paired values. Intergroup comparisons were made with a two-way ANOVA with the Bonferroni correction for multiple comparisons, and statistical significance was set at *P*<0.05.
