# Supplementary material for: BBC3 in macrophages promoted pulmonary fibrosis development through inducing autophagy during silicosis
Source: Cell Death Dis. 2017 Mar 9;8(3):e2657–. doi: 10.1038/cddis.2017.78 (PMC5386570; doi:10.1038/cddis.2017.78)
Supplement: Supplementary Information [file cddis201778x1.docx]

**Supplementary Information**

**BBC3 in macrophages promoted** [**pulmonary**](javascript:void(0);) [**fibrosis**](javascript:void(0);) **development through inducing autophagy during silicosis**

Authors: Liu, Cheng, Yang, Wang, Fang, Zhang, Han, Zhou, Yao, Chao, Liao

**Table of Contents**

Supplementary Figure S1 2

Supplementary Figure S2 3

Supplementary Figure S3 4-5

Supplementary Figure S4 6-7

Supplementary Figure S5 8-9

Supplementary Figure S6 10-11

**Figure S1**

**
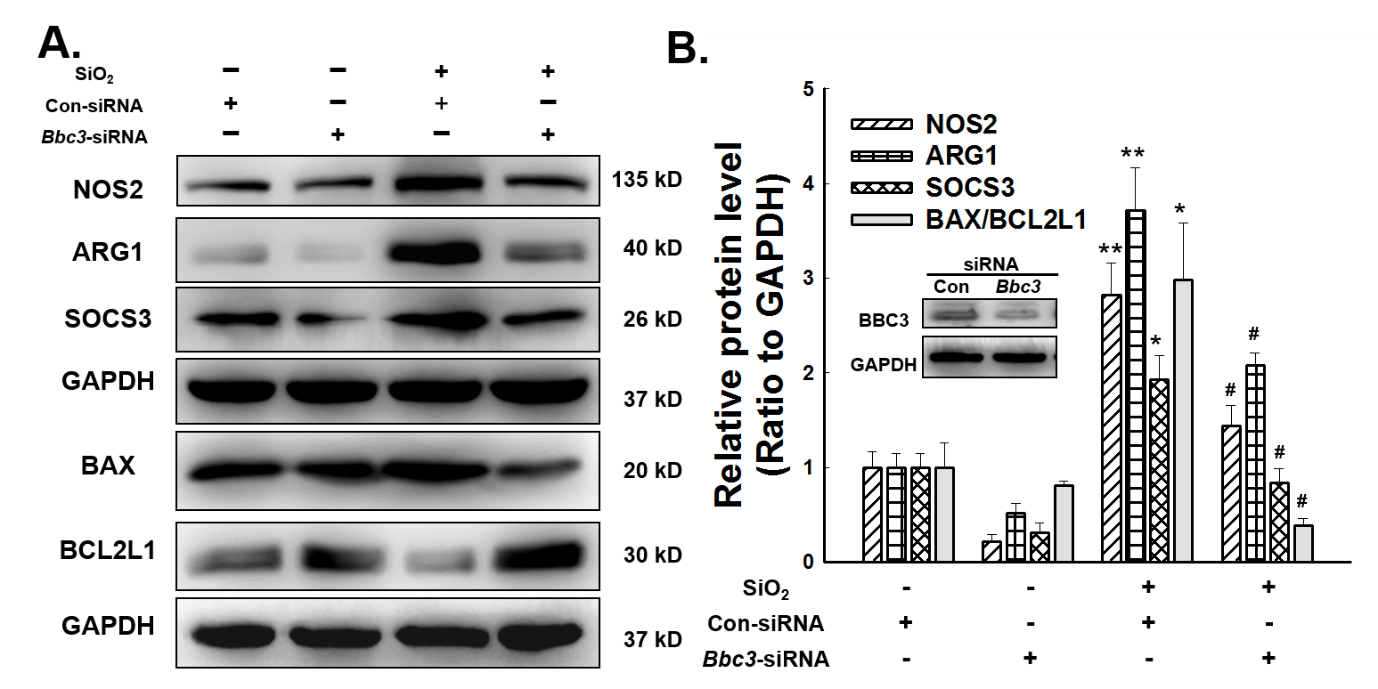
**

**Fig. S1** Increased BBC3 expression in macrophages induced by silica exposure caused activation and apoptosis

**(A-B)** Representative Western blot and densitometric analyses showing the effects of *Bbc3*-specific siRNA on SiO_2_-induced NOS2, ARG1, SOCS3, BAX and BCL2L1 expression in U937 cells. *Bbc3*-specific siRNA attenuated the increase in NOS2, ARG1, SOCS3 and BAX/BCL2L1 expression induced by SiO_2_. Data are presented as the mean ± SEM (n =5); * p<0.05; ** p<0.01 vs the con-siRNA group; # p<0.05 vs the con-siRNA+SiO_2_ group (two-way ANOVA).

**Figure S2**


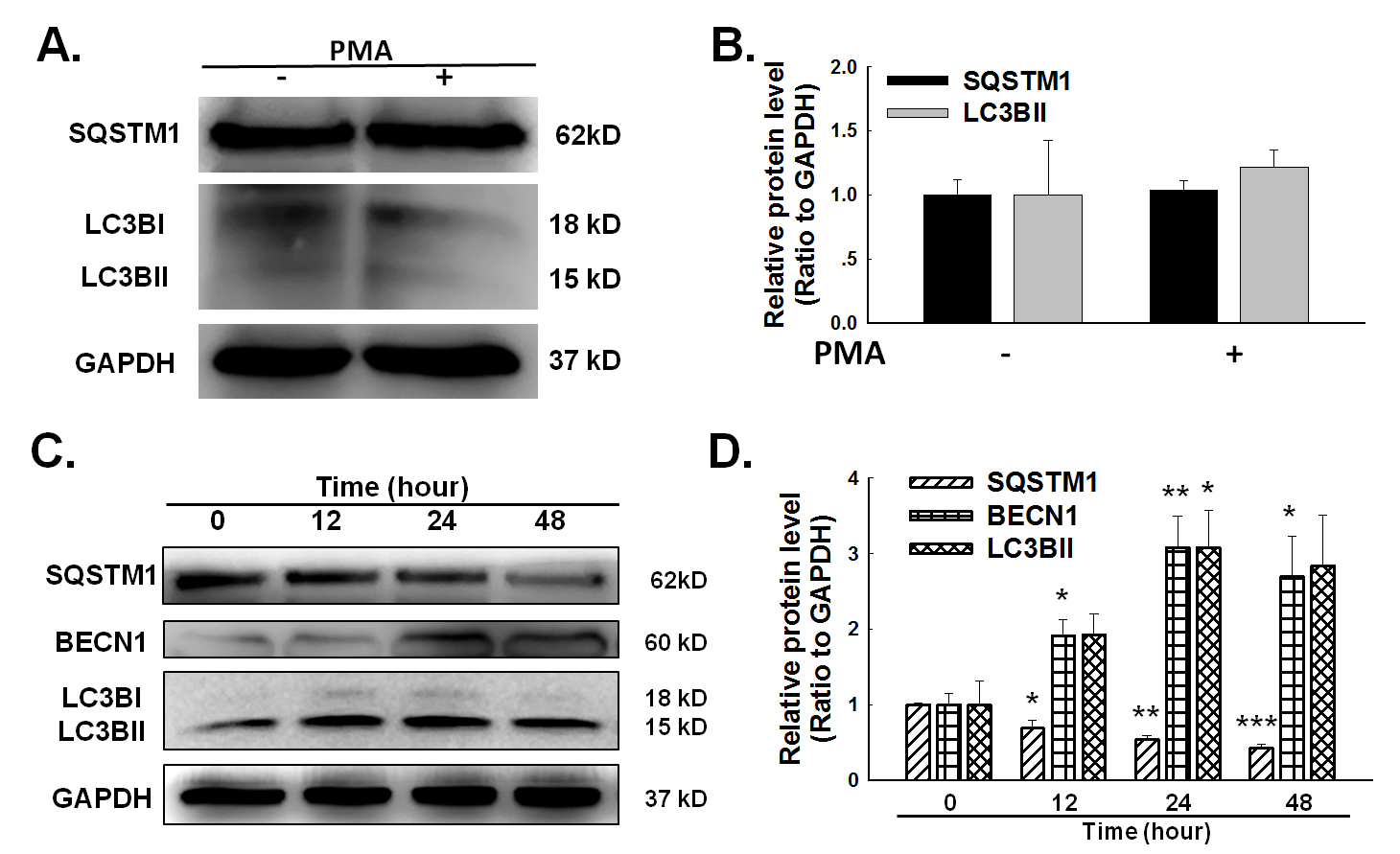


**Fig. S2** SiO_2_ upregulated the marker protein of autophagy in macrophages exposed to silica

**(A-B)** PMA (Phorbol myristate acetate) (50 nM) was used to differentiate U937 cells for 24 hours. Then the PMA was removed and the U937-differentiated macrophages were cultured for another 24h prior to the experiments. Representative Western blot and densitometric analyses showing that PMA had no significant effects on expression of LC3BII and SQSTM1. Data are presented as the mean ± SEM (n = 3).

**(C-D)** Representative Western blot and densitometric analyses showing that SiO_2_ induced the expression of the autophagy markers BECN1 and LC3BII, but reduced the expression of SQSTM1 in U937 cells in a time-dependent manner. Data are presented as the mean ± SEM (n = 5); * p<0.05; ** p<0.01; *** p<0.001 vs the 0-h group (Student’s t-test).

**Figure S3**


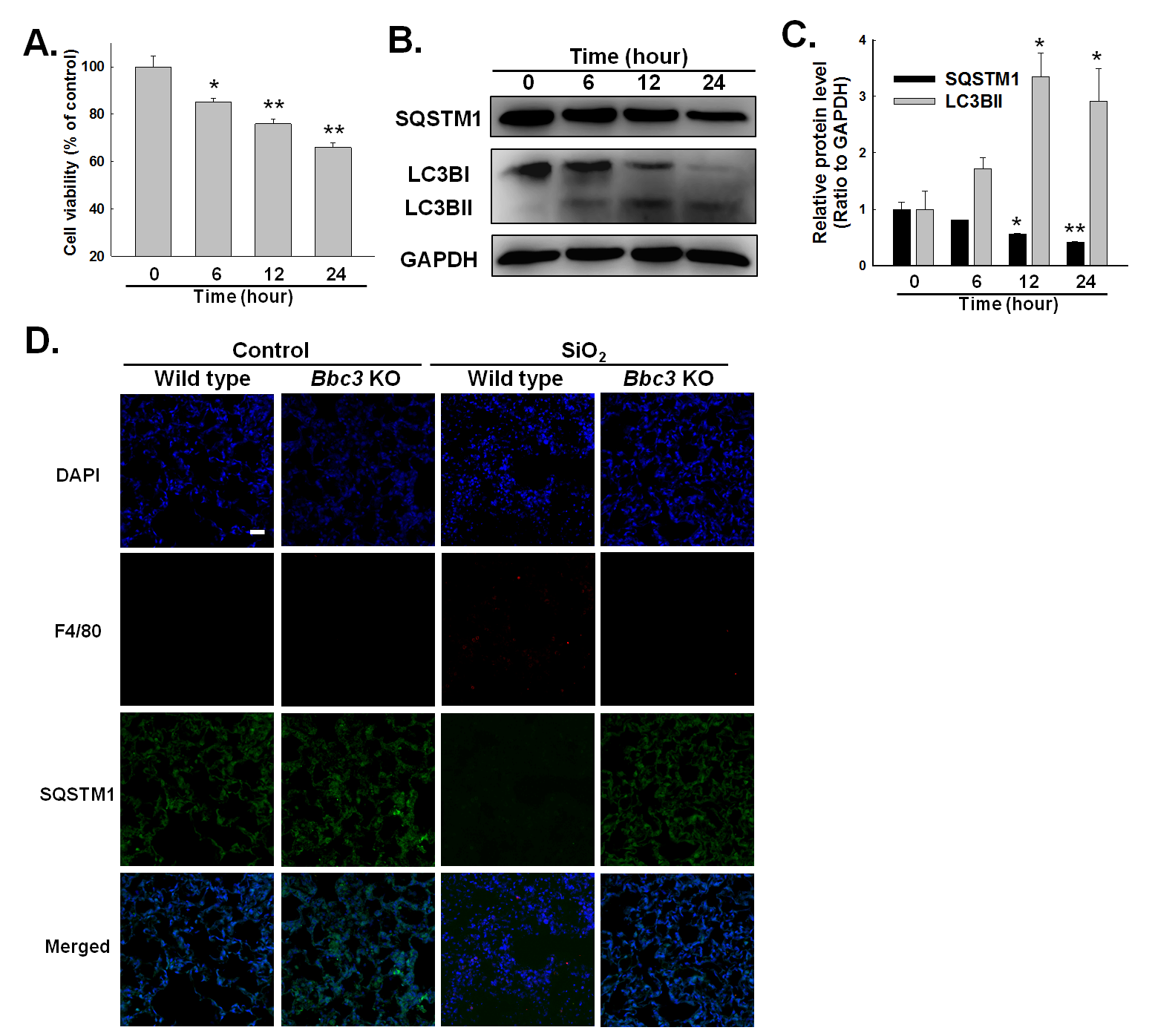


**Figure S3** Autophagy was involved in BBC3-mediated silicosis development

**(A)** MTT assay results showing that the SiO_2_-induced decrease in cell viability occurred in a time-dependent manner in bone marrow-derived macrophages (BMDMs). Data are presented as the mean ± SEM (n=5); * p<0.05; * *p<0.01 vs the 0-h group (Student’s t-test). **(B-C)** Representative Western blot and densitometric analyses showing that SiO_2_ induced the expression of the LC3BII, but reduced the expression of SQSTM1 in BMDMs in a time-dependent manner. Data are presented as the mean ± SEM (n = 3); * p<0.05; ** p<0.01 vs the 0-h group (Student’s t-test). **(D)** Immunohistochemical staining of WT and *Bbc3* KO mouse lung tissue showing autophagy intensity. The results indicated that the loss of BBC3 reduced the number of macrophages and increased the expression of SQSTM1 in lung tissue sections. Scale bar=20 μm. Images are representative of several individuals from each group (n=4).

**Figure S4**


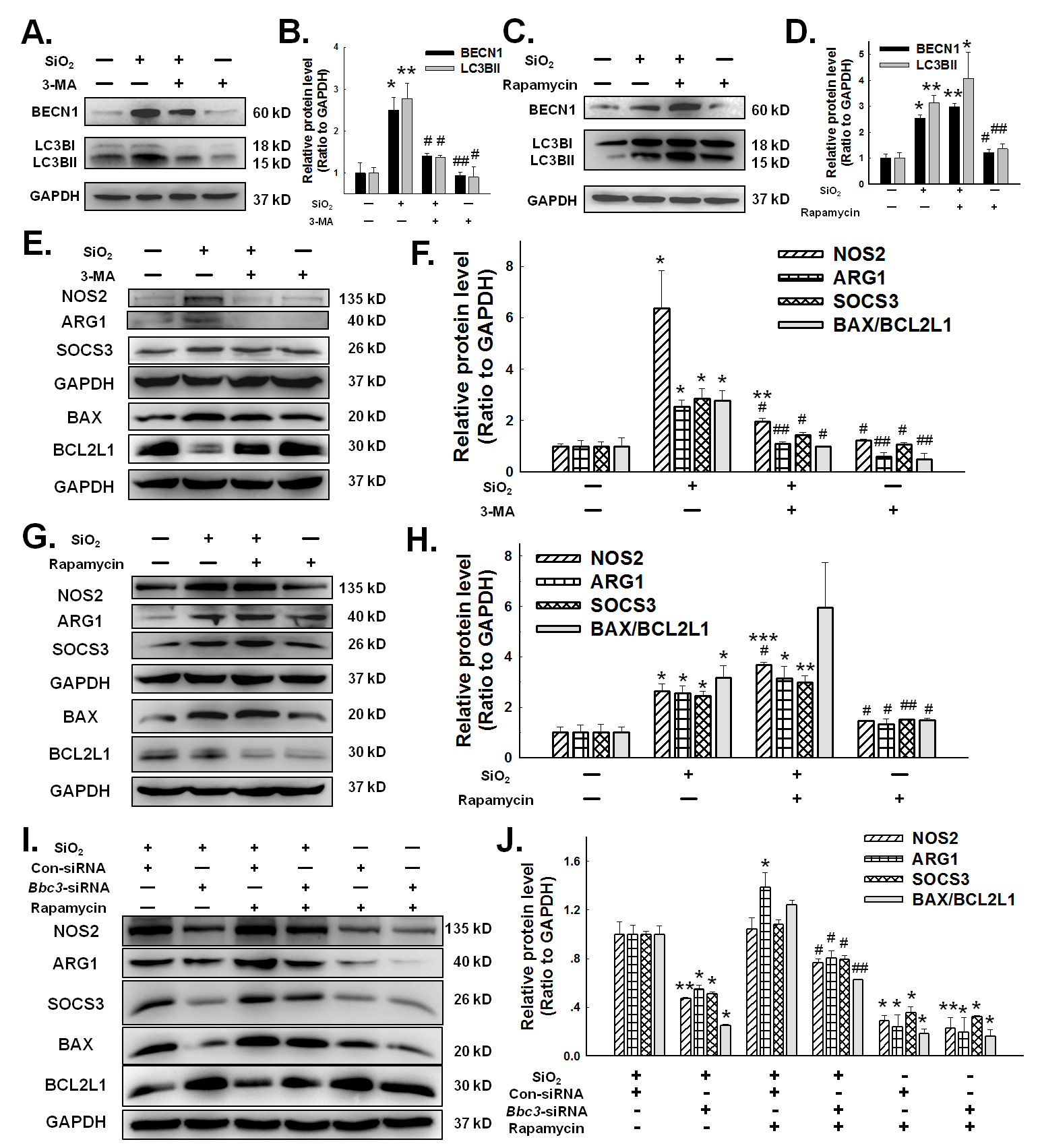


**Fig. 4** Autophagy mediated the activation and apoptosis of macrophages exposed to silica

**(A-B)** Representative Western blot and densitometric analyses showing the effects of 3-MA (1 mM), an inhibitor of autophagy, on the expression of BECN1 and LC3B in U937 cells; 3-MA significantly inhibited the expression of BECN1 and LC3BII induced by SiO_2._ **(C-D)** Representative Western blot and densitometric analyses showing the effects of rapamycin (1 μM), an inducer of autophagy, on the expression of BECN1 and LC3B in U937 cells; rapamycin further enhanced the expression of BECN1 and LC3BII induced by SiO_2_. Representative Western blot and densitometric analyses showing the effects of 3-MA and rapamycin on the expression of NOS2, ARG1, SOCS3, BAX and BCL2L1; 3-MA significantly inhibited the expression of NOS2, ARG1, SOCS3, BAX/ BCL2L1 induced by SiO_2_ **(E-F)**, but rapamycin further enhanced the effects of SiO_2_ on U937 cells **(G-H)**. Data are presented as the mean ± SEM (n=5); * p<0.05; ** p<0.01 vs the control group; # p<0.05; ## p<0.01 vs the SiO_2_ group (two-way ANOVA). **(I-J)** Representative Western blot and densitometric analyses showing the effects of rapamycin and *Bbc3*-specific siRNA on the SiO_2_-induced expression of NOS2, ARG1, SOCS3, BAX and BCL2L1 in U937 cells. Rapamycin reversed the effects of *Bbc3*-specific siRNA on NOS2, ARG1, SOCS3 and BAX/BCL2L1 expression induced by SiO_2_. Data are presented as the mean ± SEM (n=5). * p<0.05; ** p<0.01 vs the con-siRNA+SiO_2_ group; # p<0.05; ## p<0.01 vs the *Bbc3*-siRNA+SiO_2_ group (two-way ANOVA).

**Figure S5**

**
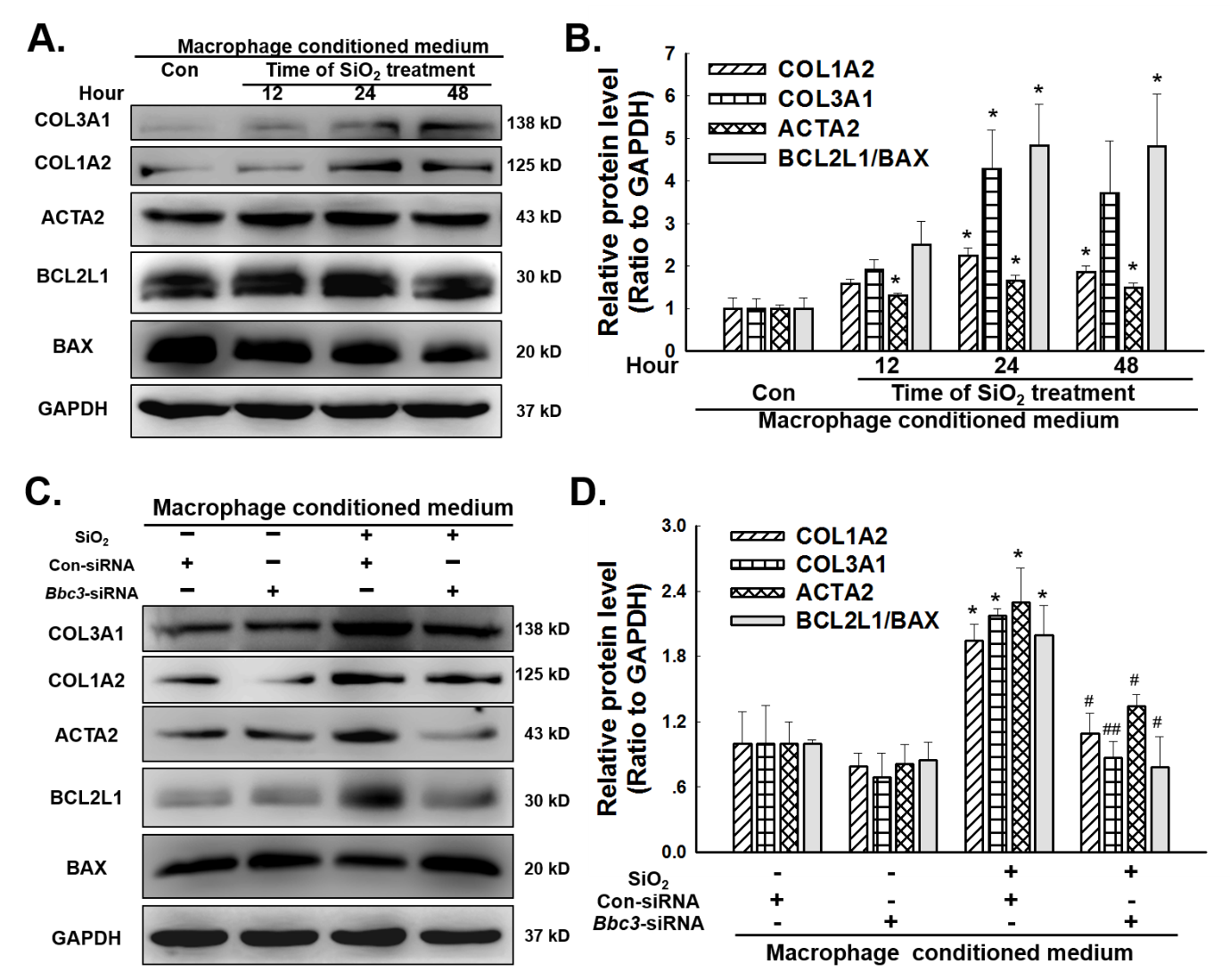
**

**Fig. S5** BBC3 up-regulation in macrophages mediated conditioned medium-induced pro-fibrotic phenotype

**(A-B)** Representative Western blot and densitometric analyses showing the effects of macrophage-conditioned medium following treatment with SiO_2_ on COL1A2, COL3A1, ACTA2, BAX and BCL2L1 expression in HPF-a cells. The results suggested that the macrophage-conditioned medium from the SiO_2_ group increased COL1A2, COL3A1, ACTA2 and BCL2L1/BAX expression in HPF-a cells. Data are presented as the mean ± SEM (n=5); * p<0.05 vs the 0-h group (Student’s t-test). **(C-D)** Representative Western blot showing the effects of conditioned medium from U937 cell-derived macrophages treated with *Bbc3*-specific siRNA on COL1A2, COL3A1, ACTA2, BAX and BCL2L1 expression in HPF-a cells. *Bbc3*-specific siRNA attenuated the increase in COL1A2, COL3A1, ACTA2 and BCL2L1/BAX expression induced by SiO_2_. Data are presented as the mean ± SEM (n=5); * p<0.05 vs the con-siRNA group; # p<0.05; ## p<0.01 vs the con-siRNA+SiO_2_ group (two-way ANOVA).

**Figure S6**

**
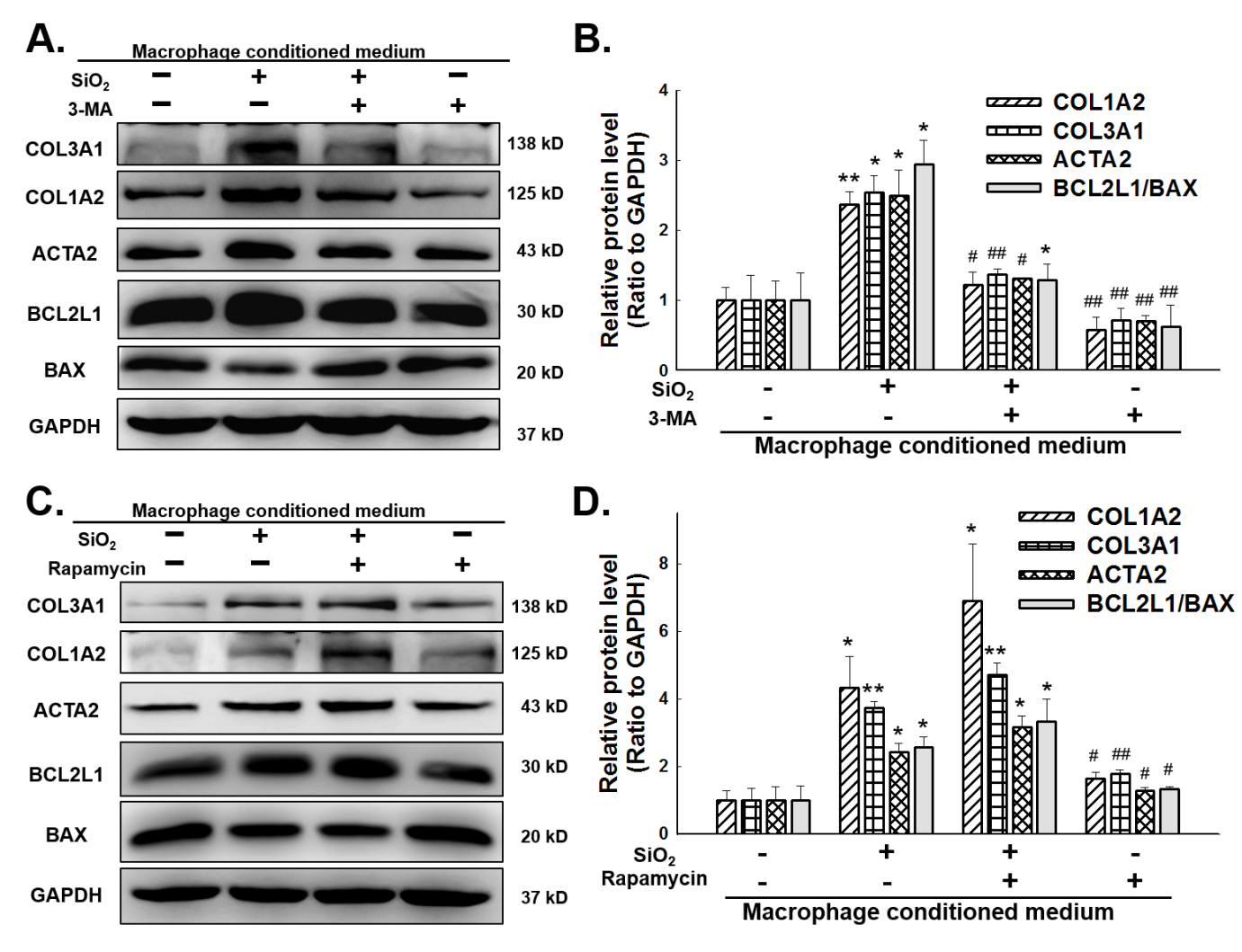
**

**Fig. S6** Autophagy in macrophages exposed to silica mediated the fibrotic effects of conditioned medium on HPF-a cells

**(A-B)** Representative Western blot and densitometric analyses showing the effects of the macrophage-conditioned medium on the expression of COL1A2, COL3A1, ACTA2, BAX and BCL2L1 in HPF-a cells. The pretreatment of U937 cells with 3-MA attenuated the increase in COL1A2, COL3A1, ACTA2 and BCL2L1/BAX expression induced by the conditioned medium of macrophages treated with SiO_2_. **(C-D)** However, the pretreatment of U937 cells with rapamycin further enhanced the pro-fibrotic effects of macrophage-conditioned medium induced by SiO_2_. Data are presented as the mean ± SEM (n=5); * p<0.05; ** p<0.01 vs the control group; # p<0.05; ## p<0.01 vs the SiO_2_ group (two-way ANOVA).
